# A High-Sensitivity Vacuum Diode Temperature Sensor Based on Barrier-Lowering Effect

**DOI:** 10.3390/mi13020286

**Published:** 2022-02-10

**Authors:** Zhihua Shen, Xiao Wang, Qiaoning Li, Bin Ge, Linlin Jiang, Jinshou Tian, Shengli Wu

**Affiliations:** 1 School of Electronics and Information Engineering, Nantong Vocational University, Nantong 226007, China; 9000082@mail.ntvu.edu.cn (Q.L.); Bge@mail.ntvu.edu.cn (B.G.); jllllj18818262694@163.com (L.J.); 2 School of Electronic Information and Artificial Intelligence, Shaanxi University of Science and Technology, Xi’an 710049, China; wangxiao@sust.edu.cn; 3 State Key Laboratory of Transient Optics and Photonics, Xi’an Institute of Optics and Precision Mechanics of CAS, Xi’an 710119, China; tianjs@opt.ac.cn; 4 Key Laboratory for Physical Electronics and Devices of the Ministry of Education, Xi’an Jiaotong University, Xi’an 710049, China

**Keywords:** vacuum diode, electric field assisted thermionic emission, temperature sensor

## Abstract

A new kind of temperature sensor based on a vacuum diode was proposed and numerically studied in this paper. This device operated under different electron emission mechanisms according to the electron density in the vacuum channel. The temperature determination ability of this device was only empowered when working in the electric-field-assisted thermionic emission regime (barrier-lowering effect). The simulated results indicated that the temperature-sensing range of this device was around 273 K–325 K with a supply current of 1 μA. To obtain a linear dependency of voltage on temperature, we designed a proportional-to-absolute-temperature (PTAT) circuit. The mathematic derivation of the PTAT voltage is presented in this study. The temperature-sensing sensitivity was calculated as 7.6 mV/K according to the measured I-U (current versus voltage) characteristic. The structure and principle of the device presented in this paper might provide an alternative method for the study of temperature sensors.

## 1. Introduction

Temperature sensors play an important role in many fields, such as industrial automation, medical monitoring, food safety, and portable electronic devices [1,2,3,4]. In the continuous pursuit of miniaturization, temperature sensors with the merits of low cost, low power consumption and compatibility with integrated circuits, attract much attention [5,6,7]. Among these different types of temperature sensors, semiconductor-based diodes or transistor sensors are the most common devices due to their high sensitivity and full compatibility with complementary metal oxide semiconductor (CMOS) technology [8]. To achieve a high sensitivity and wider temperature range, extensive studies were conducted, focusing on the Schottky diode [8,9,10,11,12,13,14], p-n junction diode [15,16] and p-i-n diode [17,18,19]. The sensitivity of these sensors varied from 0.61 mV/K [18] to 5.11 mV/K [8] and the operating temperature limit reached 440 K [10].

Recently, many studies on vacuum electronic devices were conducted, involving vacuum diodes and triodes [20,21,22], where the vacuum channel length was designed at the nanometer scale. Thus, these devices can operate in air without performance degradation. As is known to all, a vacuum is the superior media for carrier transportation, as opposed to semiconductor materials, where electrons travel ballistically without being scattered by a lattice or captured by defects. Vacuum electronics are generally considered to be more suitable for harsh environments [20,21,22] or faster detecting [23] than solid-state electronics. The existing vacuum devices are used for photon detection [23] or signal modulation [24,25]. To the best of our knowledge, the application of vacuum diodes or triodes for temperature sensing has not yet been studied.

In this work, we proposed a nanoscale vacuum diode temperature sensor based on electric-field-assisted thermionic emission. The sensitivity reached 7.6 mV/K (calculated according to the I-V curve measured in [26]), which is higher than 5.11 mV/K, as reported in [8]. The finite integration technique was adopted to numerically study the performance of the diode on temperature-sensing ability. Moreover, a PTAT circuitry based on this vacuum diode was also proposed, and the corresponding mathematical analytic scheme was elaborated. The temperature detecting sensitivity of a single diode and the PTAT circuitry was calculated.

## 2. Methods

### 2.1. Device Structure and Working Principle

**Figure 1:**
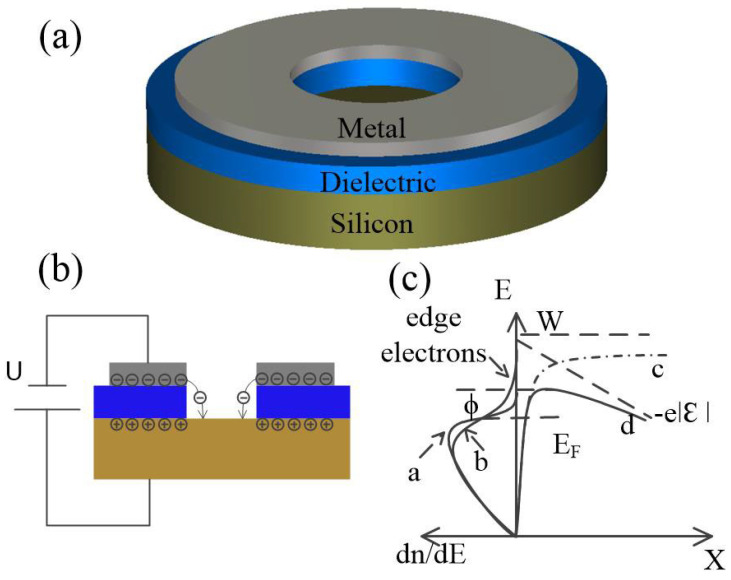
The vacuum diode temperature-sensing element. (**a**) Schematic of the proposed temperature-sensing diode; (**b**) Schematic illustration of electrons emission and transportation in the vacuum channel. Electrons will be ejected from the edge of 2DES, travelling from metal to silicon via vacuum channel; (**c**) Diagram of electron distribution by energy in cathode and surface barrier distribution. W is the height of surface vacuum barrier. E_F_ is the Fermi level of cathode. Line a is normal electron distribution in metal, which we deliberately show here as a reference. Line b is electron distribution in metal, where 2DES developed under certain bias. Line c is surface barrier distribution of electrons without external electric field. Line d is surface barrier distribution of electrons under external electric field.

**Figure 2:**
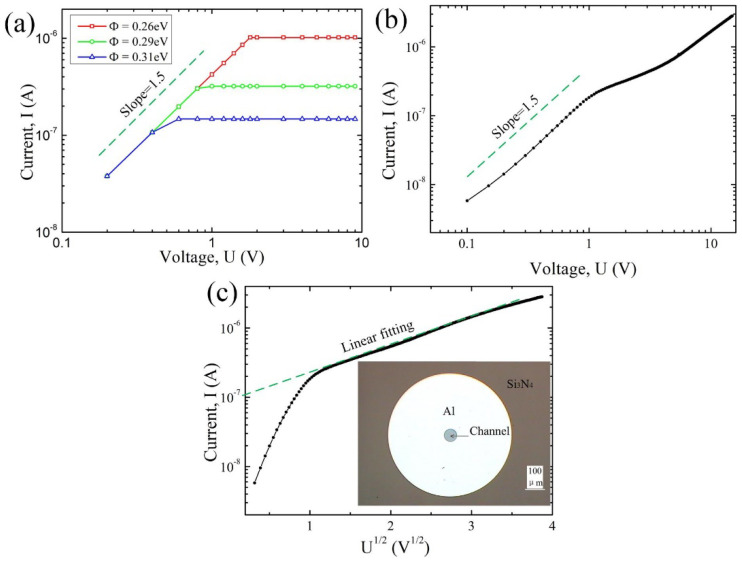
I−U curve of the proposed vacuum diode under room temperature. (**a**) Simulated results of I−U characteristics with different equivalent work functions. (**b**) Measured I−U characteristics in a lg−lg plot. (**c**) Measured I−U characteristics in a lg I−U^1/2^ plot. The inset is a picture of the diode taken by metallographic microscope. The linear relationship indicated the barrier−lowering effect mechanism.

The proposed temperature-sensing element is a vertically aligned MIS diode with a void channel in the center, as illustrated in Figure 1a. The cathode material is aluminum. Silicon substrate (boron-doped, resistivity = 10 Ωcm, (100)-oriented) acts as anode. The dielectric layer material is silicon nitride (Si_3_N_4_), and the thickness is 80 nm (comparable to the mean free path of electrons in air). The radius of the vacuum channel is set as 30 μm. Electrons accumulate at the cathode–dielectric interface forming the so-called quasi-two-dimensional electron system (2DES) (width <1 nm) [24,27] in the cathode side under negative voltage bias, as shown in Figure 1b. In the 2DES, electrons can travel freely in the lateral dimension but can hardly move in the vertical dimension. Electrons near the cleaved edge of the void channel suffer asymmetrical Coulombic repulsion from surrounding electrons and are readily injected into the vacuum channel. Thus, the surface vacuum barrier height of the 2DES is much lower than the work function of Al (4.28 eV). Moreover, the height of surface barrier will further decrease under external electric field bias, as depicted in Figure 1c. When the voltage bias was relatively low (<1 V), emission current I of this device was subjected to space-charge limitation (SCL) regime, where the current exhibited a 3/2 power dependency of voltage and was irrelevant to temperature, as demonstrated in our former work [28]. As the voltage bias increased, the electron emission mechanism then turned into the barrier-lowering effect regime (field-assisted thermionic emission), where lg I showed a linear relationship with *U*^1/2^/*T*, *U* refers to bias voltage and *T* refers to temperature, as shown in Figure 2c and Equation (1) which will be discussed later. The experimental fabrication details and verification of the electron emission mechanism can be seen in the Supplementary Materials in our former work [26,28].

### 2.2. Simulation Technique

To investigate the temperature-sensing ability of the vacuum diode, CST PARTICLE STUDIO^TM^, an electromagnetic field and particle-tracking simulation software based on finite integration technique (FIT), was used. The current density of the emission was calculated by Richardson–Dushman equation, *j* = *AT*^2^exp(−Φ/k*T*), where *A* is the emission constant and set as the default value of 1.2 × 10^6^ A·m^−2^·K^−2^, Φ is the equivalent work function of cathode, and k is Boltzmann’s constant. The width of the emission surface was set as 1 nm according to [24]. Due to the barrier-lowering effect, Φ decreases as the external electric field increases. The equivalent work function Φ under a different voltage bias will be determined in advance using the data measured under room temperature by Keithley 4200. Notably, the measurement data mentioned in the following article are all derived from [28].

## 3. Results and Discussion

Two different electron emission mechanism were observed, as shown in Figure 2. Under a relatively low voltage bias, the electron emission mechanism was subjected to the space-charge limited current regime, as shown in Figure 2a,b, which was dominated by the virtual cathode. As the voltage bias increased, the virtual cathode was pushed towards the cathode and eventually landed on it. Meanwhile, the barrier-lowering effect emerged, as shown in Figure 2c. The equivalent work function of the cathode decreased continuously with the increase in voltage bias. Thus, the emission current increased correspondingly. The inflection point of the current in the I-U curve was related to the equivalent work function as shown in Figure 2a. As Φ was a constant in the space-charge limited regime; we could easily calculate it by a tentative simulation. By comparing the simulated results and measured data, as shown in Figure 2a,b, Φ in the space-charge limited regime was determined as 0.29 eV, where the voltage bias corresponding to the inflection point of the current was around 1 V. The barrier-lowering effect was intentionally ignored in Figure 2a for simplification.

**Figure 3:**
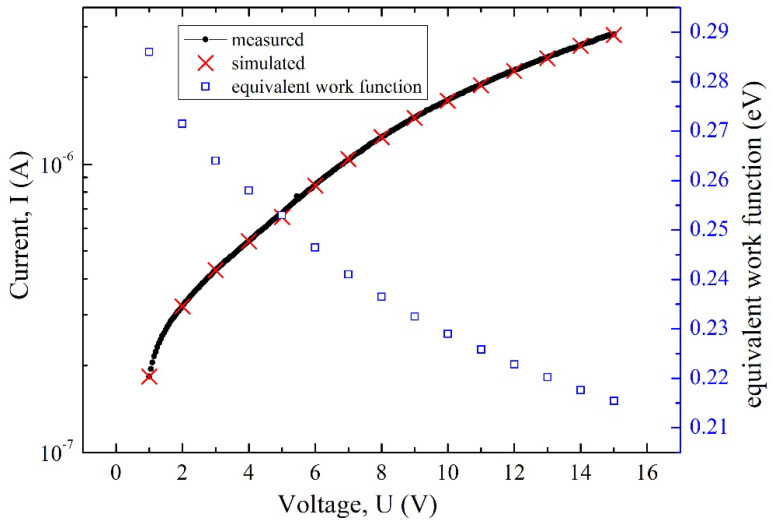
Equivalent work function of cathode under different voltage bias.

As for the barrier-lowering effect regime, the surface barrier of the cathode decreased as the voltage bias increased, as mentioned above. The work function in the Richardson–Dushman equation could be treated as equivalently decreased during the calculation of the emission current. Hence, the equivalent work function corresponding to the applied voltage bias could be calculated from the measured data, considering that the practical electron emission surface was not ideal as the simulating model. Additionally, the actual emission surface might be speckled, and the area would be smaller than the calculated value of the model. The effective electron emission area was then calibrated with a coefficient of 1.74 according to the measured data. The equivalent work function could be uniquely determined when the simulated results fitted the experimental results measured at room temperature, as depicted in Figure 3. If a constant current source was applied to the diode, then temperature was a function of Φ according to the Richardson–Dushman equation. In other words, the voltage bias was related to temperature. Therefore, this diode could be used as a temperature sensor.

**Figure 4:**
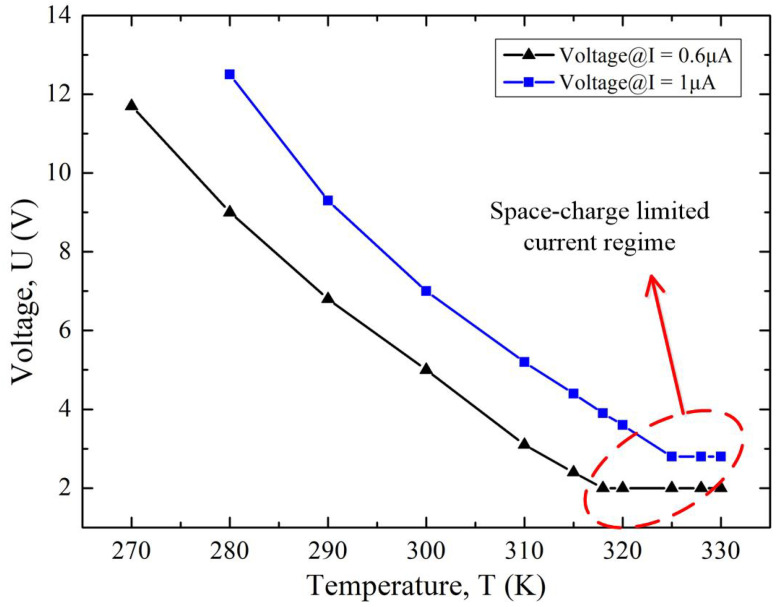
Simulated temperature response characteristics of the diode.

Figure 4 showed the simulated temperature response characteristics of the diode under two different constant current values. In the barrier-lowering effect regime, voltage was negatively related to temperature. Meanwhile, voltage was irrelevant to temperature if the diode was operated in the space-charge limited current regime, in which the electric field was too weak to attract the emitted electrons to the anode in time, forming the so-called virtual cathode in the vacuum channel. Hereby, the current was governed by voltage rather than temperature. In order to ensure that this diode was working in the barrier-lowering effect regime, the upper limit of the temperature-sensing range was confined, as shown in Figure 4. Considering the power consumption issue, the lower limit of the temperature-sensing range was also confined. The effective sensing range could be modified by the constant current source, as depicted in Figure 4. According to Figure 4, the temperature-sensing sensitivity of a single diode could be estimated to be 200 mV/K.

**Figure 5:**
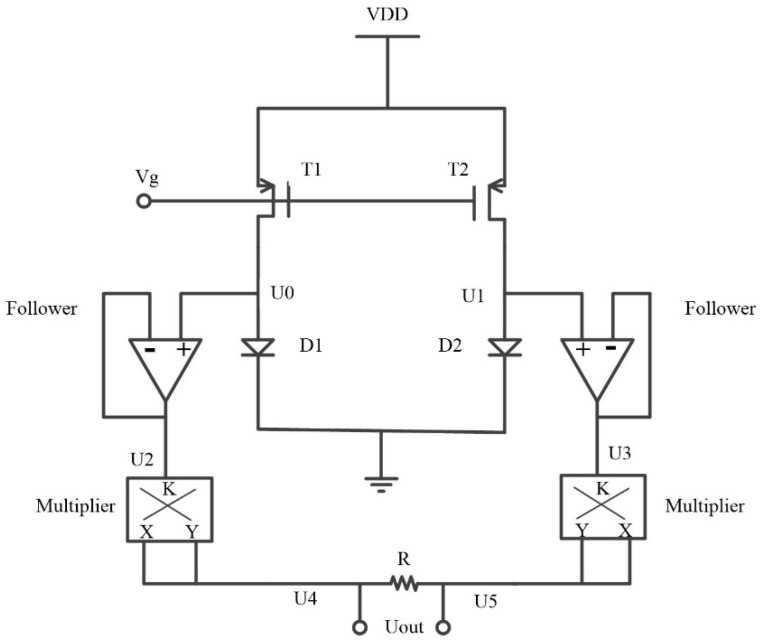
Schematic of the proposed PTAT circuitry.

However, according to Figure 4 an Equation (1), the relationship between the voltage and temperature of the diode is nonlinear. To achieve a linear dependency of voltage on temperature, a PTAT circuitry was proposed, as shown in Figure 5. Transistors T1 and T2 form a current mirror, supplying exactly same current to vacuum diodes D1 and D2. The emission area of D1 and D2 met the relationship of D1 = *ρ*D2 (*ρ* > 1). The stack layer materials and vacuum channel length of the two diodes are exactly the same. The current emission of the diodes satisfies the following equations:

(1) $$\mathrm{lg}I_{1}=\mathrm{lg}I_{01}+\beta \sqrt{U_{0}}/T$$

(2) $$\mathrm{lg}I_{2}=\mathrm{lg}I_{02}+\beta \sqrt{U_{1}}/T$$

where *I*_1_ and *I*_2_ are current through the diodes, *β* is a constant related to the material and geometric dimensions of the diodes, *I*_01_ and *I*_02_ are the emission current at zero electric field. According to the relationship of lg *I* and *U*^1/2^ in Figure 1c, the value of *β* reflects the slope of the curve and can be calculated as 125, where T = 300 K. In the equations, *I*_1_ = *I*_2_ and *I*_01_ = *ρI*_02_. Subtracting Equation (1) from Equation (2) and expressing it in terms of temperature *T*, we obtain Equation (3) in the form:

(3) $$T=\frac{\beta \left(\sqrt{U_{1}}-\sqrt{U_{0}}\right)}{\mathrm{lg}\rho }$$

where absolute temperature *T* is proportional to $\sqrt{U_{1}}-\sqrt{U_{0}}$. According to Figure 5, the following equations are obtained as:

(4) $$U_{2}=U_{0}\;U_{3}=U_{1}\;U_{4}=\sqrt{\frac{1}{K}}\cdot \sqrt{U_{2}}\;U_{5}=\sqrt{\frac{1}{K}}\cdot \sqrt{U_{3}}\;U_{out}=U_{5}-U_{4}$$

where *K* is the gain coefficient of multiplier (typical value is 0.1 V^−1^). Then, *U*_out_ can be expressed as:

(5) $$U_{out}=\sqrt{\frac{1}{K}}\cdot \left(\sqrt{U_{1}}-\sqrt{U_{0}}\right)$$

Substituting Equation (5) into Equation (3) and expressing *T*, we obtain:

(6) $$T=\frac{\beta \cdot \sqrt{K}}{\mathrm{lg}\rho }\cdot U_{out}$$

where *U_out_* is the PTAT voltage. By adjusting the value of *ρ*, the temperature-sensing sensitivity seems to be adjustable. However, in order to make the working range of the two diodes close, so as to lower the supply voltage (<15 V) and maximize the temperature measurement range, the value of ρ should not be too large. Here, we set it to 2 as a typical value. Then, the temperature-sensing sensitivity of this device can be calculated as 7.6 mV/K.

## 4. Conclusions

A novel vacuum diode working in the barrier-lowering effect regime was demonstrated to have a temperature-sensing ability. In this device, the temperature and external voltage both affect the emission current density. When a constant current source is used as a power supply, the voltage and temperature of the device have a unique correspondence. However, if the voltage is not high enough, the current and voltage satisfy the space-charge current-limiting mechanism. The vacuum diode will no longer have a temperature-sensing ability. Therefore, the temperature detection range is limited to below 325 K, although it could be slightly adjusted by the current source. To achieve a linear dependency of temperature on voltage, a PTAT circuit was proposed. The mathematical derivation of the PTAT voltage was also demonstrated. The temperature-sensing sensitivity of this device was calculated to be 7.6 mV/K. As a vacuum diode, electrons are transported in a vacuum rather than semiconductor layers, which allows to become a high-quality temperature sensor. This work provided a new direction for the research on novel temperature sensors. Further study on widening the temperature-sensing range and device fabrication will be carried out in our later work.
